# Effects of *Eurycoma longifolia* on Testosterone Level and Bone Structure in an Aged Orchidectomised Rat Model

**DOI:** 10.1155/2012/818072

**Published:** 2012-08-26

**Authors:** Abdul Shukor Tajul Ariff, Ima Nirwana Soelaiman, J. Pramanik, Ahmad Nazrun Shuid

**Affiliations:** ^1^Kulliyyah of Medicine and Health Sciences, Insaniah University College, Lebuhraya Sultanah Bahiyah, 05350 Alor Setar, Malaysia; ^2^Department of Pharmacology, Faculty of Medicine, Universiti Kebangsaan Malaysia, Jalan Raja Muda Abdul Aziz, 50300 Kuala Lumpur, Malaysia; ^3^Institute of Research & Post Graduate Studies, Allianze University College of Medical Sciences, Waziria Medical Square, 13200 Kepala Batas, Malaysia

## Abstract

Testosterone replacement is the choice of treatment in androgen-deficient osteoporosis. However, long-term use of testosterone is potentially carcinogenic. *Eurycoma longifolia* (EL) has been reported to enhance testosterone level and prevent bone calcium loss but there is a paucity of research regarding its effect on the bone structural parameters. This study was conducted to explore the bone structural changes following EL treatment in normal and androgen-deficient osteoporosis rat model. Thirty-six male Sprague-Dawley rats aged 12 months were divided into normal control, normal rat supplemented with EL, sham-operated, orchidectomised-control, orchidectomised with testosterone replacement, and orchidectomised with EL supplementation groups. Testosterone serum was measured both before and after the completion of the treatment. After 6 weeks of the treatment, the femora were processed for bone histomorphometry. Testosterone replacement was able to raise the testosterone level and restore the bone volume of orchidectomised rats. EL supplementation failed to emulate both these testosterone actions. The inability of EL to do so may be related to the absence of testes in the androgen deficient osteoporosis model for EL to stimulate testosterone production.

## 1. Introduction

Hormonal replacement therapy with testosterone is the choice of treatment for hypogonadism in men [1–3]. It is well documented that testosterone therapy improves bone mineral density (BMD) and promote patient compliance in such cases [1, 4]. However, long-term use of testosterone is potentially carcinogenic [1, 5] and commonly causes secondary osteoporosis [6]. A suitable substitute for testosterone is needed, but there is paucity in studies discovering new alternative for the treatment of osteoporosis secondary to hypogonadism.

Several studies have shown androgen-like or testosterone-enhancing effects of *Eurycoma longifolia *(EL). These were evident in animal studies where EL has increased the copulatory activity, levator ani muscle activity, initiation of sexual performance and spermatogenesis [7–13]. Research studies on human subjects suffering from late-onset hypogonadism have shown that EL improved the patient's symptoms as well as the concentration of the testosterone serum [14]. These experimental outcomes revealed the potential of EL as a natural substitute to testosterone. 

EL (Simaroubaceae family) is a traditional medical plant, known locally in Malaysia as tongkat Ali, tung saw in Thailand, pasak bumi in Indonesia, and cay ba bihn in Vietnam [15]. The root has been proven to have medicinal values, which is rich in various bioactive compounds (eurycomaoside, eurycolactone, eurycomalactone, eurycomanone, and pasak bumi-B), among which the alkaloids and quassinoids form a major portion [15, 16]. The standardized water soluble extract of EL has also exhibited antimalarial properties and anticancer effects [17–19]. A recent study showed that EL can prevent bone calcium loss in orchidectomised rats (osteoporotic animal model) and has the potential as an alternative treatment for androgen-deficient osteoporosis [20]. This beneficial effects on bone may have been contributed by its testosterone-enhancing effects. To date, there has been no published report regarding the effect of EL on the bone structural parameters. With the current scenario that EL is taken by normal individual as a health supplement, the first part of the study was conducted to determine the effects of EL on the testosterone level and bone structure of normal male rat. The second part of the study was carried out to determine the effects of EL on the bone structural changes and testosterone level of androgen-deficient osteoporosis rat model.

## 2. Material and Methods

Thirty-six male Sprague-Dawley rats aged 12 months and weighing 370–500 g were used for the studies. For the first part of the study, twelve rats were divided into two groups; normal control (NC) and normal and supplemented with EL (EL) groups. For the second part of the study, the rest of the rats were divided into sham-operated (Sham), orchidectomised-control (OrxC), orchidectomised and given testosterone replacement (Orx + T) and orchidectomised and supplemented with EL (Orx + EL) groups. Before the start of the study, the rats were allowed to adjust to the new environment at least for a week. Two rats were placed in each plastic cage at 29 ± 3°C under natural day/night cycle and were fed with commercial rat chow and tap water *ad libitum*. The study was approved by the UKM Animal Ethics Committee (PP/FAR/2008/NAZRUN/13-FEB/217-FEB-2008-FEB-2010).

The body weights of the rats were measured and recorded weekly. The rats in the first part of the study were not subjected to any surgical procedures. In the second part of the study, aged orchidectomised rats were used as the model for androgen-deficient osteoporosis [21]. The rats were anesthetised with Ketapex : Xylazil (1 : 1) and scrotal approach was used to access the testis for orchidectomy [22]. A small incision 2 mm was made at the tip of the scrotum. The tunic was opened and the testis, cauda epididymis, vas deferens, and the spermatic blood vessels were exteriorized. The blood vessels and vas deferens were then ligated with 4-0 absorbable suture. The testis and epididymis were then removed. The remaining tissue was returned into the sac. The procedure was repeated for the other testis. The skin incision was closed with a nonabsorbable suture. The rats were monitored closely to avoid postsurgical complications especially chewing of the sutures and infections [22].

EL aqueous extract was supplied by Phytes Biotek Sdn. Bhd. (Malaysia). It was extracted from the root of the plant using a patented high-pressure water extraction (US Patent no: US7, 132, 117 B2). The extract was in brownish powder form and contained bioactive 22.0% eurypeptide, 41.1% glycosaponin, and 1.6% eurycomanone. EL aqueous extract powder was dissolved in normal saline. It was given to the rats through oral gavages at the dose of 15 mg/kg rat weight daily at 9 am for 6 weeks [11]. Testosterone was purchased from TCI UK Ltd (UK). It was diluted in olive oil (Bertolli, Italy), and 8 mg/kg was injected intramuscularly once daily at 9 am for 6 weeks [23]. Blood samples were collected before and after 6 weeks of treatment for measurements of testosterone serum level. Blood samples were obtained from the retroorbital vein after anesthetising the rats with ether. After 3 hours, the blood was centrifuged at 3000 rpm for 10 minutes and the serum was stored at a temperature of −70°C. After the completion of the treatment, the rats were euthanized and the femora were dissected out. 

### 2.1. Bone Histomorphometry

Bone histomorphometry is a quantitative examination of an undecalcified bone. The bone structural changes were measured using bone histomorphometric analysis according to the American Society of Bone Mineral Research Histomorphometry Nomenclature Committee 1987 [24–26]. The undecalcified left femurs were embedded in methyl methacrylate (Osteo-Bed Bone Embedding Kit; Polysciences, USA). The femora were sectioned at 9 mm thickness using a microtome (Leica RM2155, Wetzlar, Germany). The slides were stained using a modified Von Kossa method (Von Kossa, 1974) and analyzed under Nikon Eclipse 80i microscope (Nikon Instrument Inc., USA) with an image analyzer software Pro-Plus v. 5.0 (Media Cybernetics, Silver Spring, MD, USA).

Measurements at the metaphyseal region were performed for the structural bone histomorphometry. It is located at 3–7 mm from the lowest point of the growth plate and 1 mm from the lateral cortex, excluding the endocortical region [27]. The structural parameters measured were trabecular volume (BV/TV), thickness (TbTh), number (TbN), and separation (TbSp). 

### 2.2. Statistical Analysis

The results were expressed as mean ± standard error of mean (SEM). Data analysis was performed using Statistical Package for Social Sciences software (IBM SPSS Statistics v20). Data was tested for normality using the Test of Homogeneity of Variances. One-way analysis of variance (ANOVA) followed by *post hoc *Tukey test was used to test the data for significance. 

## 3. Results 

**Figure 1:**
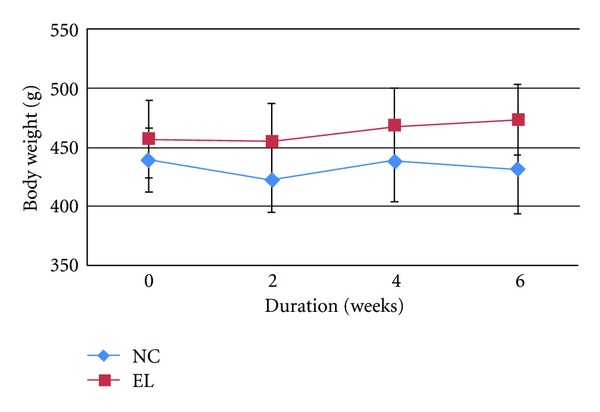
Mean body weight throughout the study. Data presented as mean ± SEM. There was no significant difference in the body weights between the groups. NC: normal control, EL: supplemented with EL.

**Figure 2:**
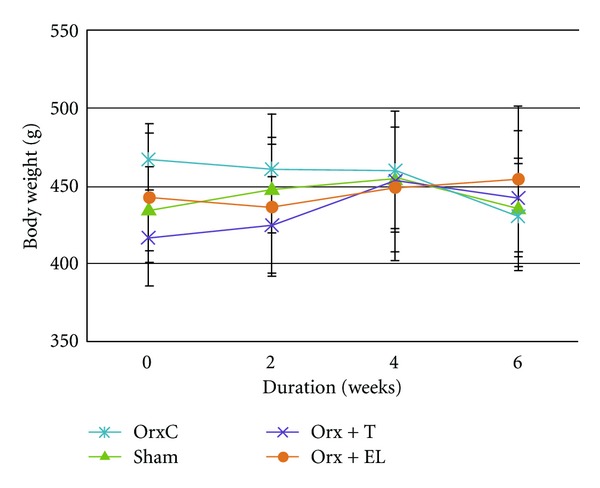
Mean body weight throughout the study. Data presented as mean ± SEM. All the groups have gained weight at the end of the study except OrxC group. There was no significant difference in the body weights between the groups. Sham: sham-operated, OrxC: orchidectomised-control, Orx + T: orchidectomised and given testosterone replacement, Orx + EL: orchidectomised and supplemented with EL.

At the end of the study, there was no significant difference in the mean body weights of the different groups throughout the study (Figures 1 and 2).

**Figure 3:**
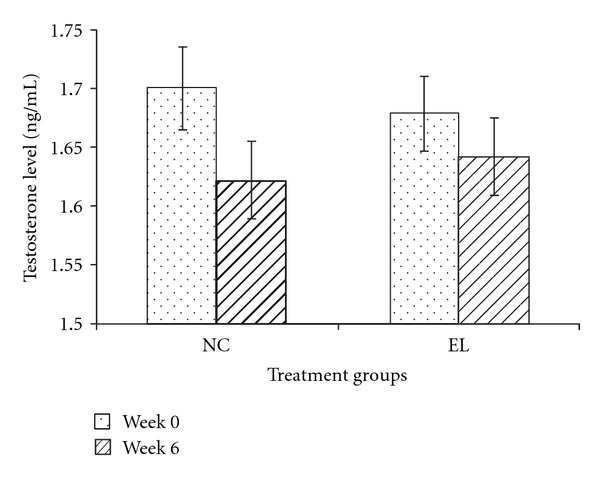
Mean testosterone level (ng/mL) at week 0 and week 6. Data presented as mean ± SEM. There was no significant difference in the serum testosterone levels between the groups. NC: normal control, EL: supplemented with EL.

**Figure 4:**
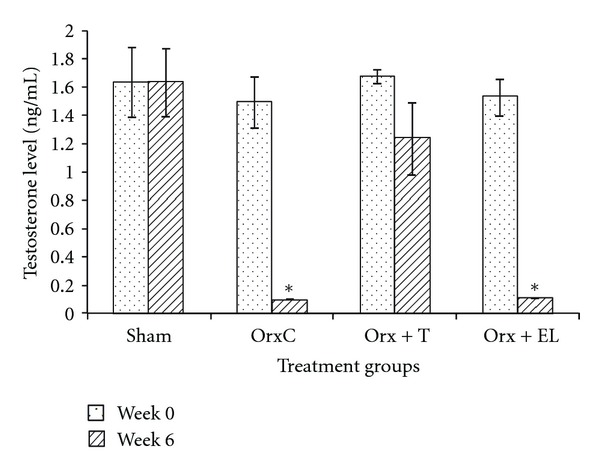
Mean testosterone level (ng/mL) at week 0 and week 6. Data presented as mean ± SEM (*P* < 0.05). *Significant difference of testosterone level at week 6 compared to Sham and Orx + T groups. Sham: sham-operated, OrxC: orchidectomised-control, Orx + T: orchidectomised and given testosterone replacement, Orx + EL: orchidectomised and supplemented with EL.

Before the treatment, there was no significant difference in the testosterone serum levels for all the groups. At the end of the 6 weeks of treatment, in the first part of the study, the testosterone level of the EL group was not significantly different from the NC group (Figure 3). In the second part of the study, the testosterone levels of the OrxC and Orx + EL groups were significantly lower than the Sham group (Figure 4). Testosterone replacement was found to elevate the testosterone level of the Orx + T group significantly higher than the OrxC and Orx + EL groups (Figure 4). 

**Figure 5:**
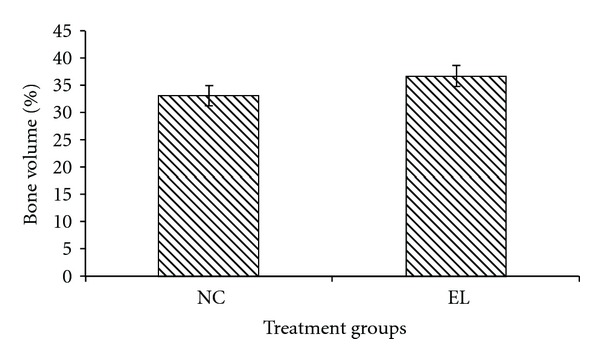
Mean trabecular bone volume for both groups. Data presented as mean ± SEM. There was no significant findings between the groups. NC: normal control, EL: supplemented with EL.

**Figure 6:**
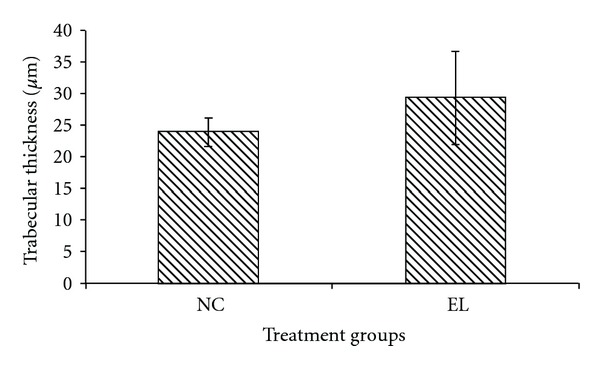
Mean trabecular thickness for both groups. Data presented as mean ± SEM. There was no significant difference in trabecular thickness between the groups. NC: normal control, EL: supplemented with EL.

**Figure 7:**
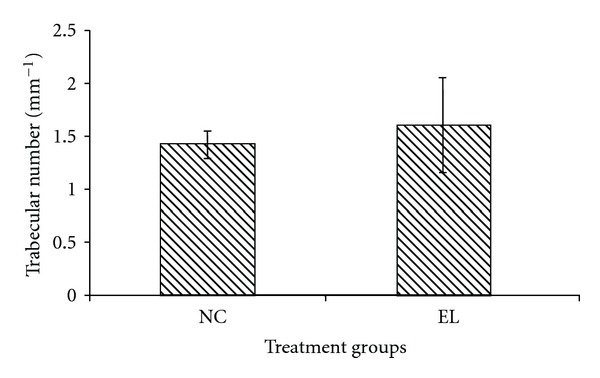
Mean trabecular number for both groups. Data presented as mean ± SEM. There was no significant difference in trabecular number between the groups. NC: normal control, EL: supplemented with EL.

**Figure 8:**
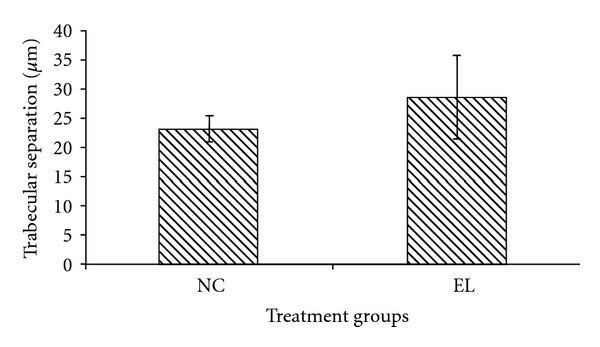
Mean trabecular separation for both groups. Data presented as mean ± SEM. There was no significant difference in trabecular separation between the groups. NC: normal control, EL: supplemented with EL.

**Figure 9:**
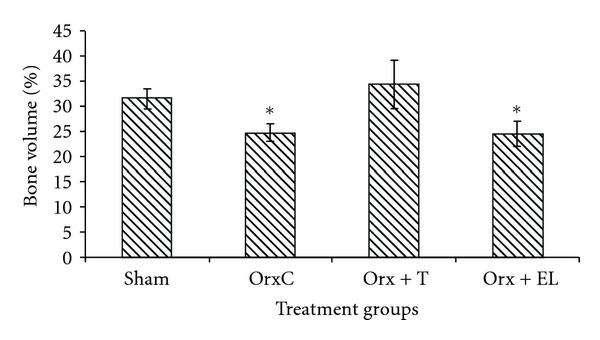
Mean trabecular bone volume for all the groups. Data presented as mean ± SEM. *Significant difference of BV/TV compared to Sham and Orx + T groups. Sham: sham-operated, OrxC: orchidectomised-control, Orx + T: orchidectomised and given testosterone replacement, Orx + EL: orchidectomised and supplemented with EL.

The bone structural parameters (BV/TV, TbTh, TbN and TbSp) were evaluated using bone histomorphometry technique. In the first part of the study, there were no significant differences in the BV/TV, TbTh, TbN, and TbSp between the NC and EL group (Figures 5, 6, 7, and 8). While for the second part of the study, orchidectomy has resulted in significantly lower BV/TV of the OrxC group compared to the Sham group. When the orchidectomised rats were given testosterone (Orx + T group), the BV/TV was restored equal to that of the Sham group. However, when the orchidectomised rats were supplemented with EL (Orx + EL), the BV/TV remained unchanged and was significantly lower than the Orx + T group (Figure 9). 

**Figure 10:**
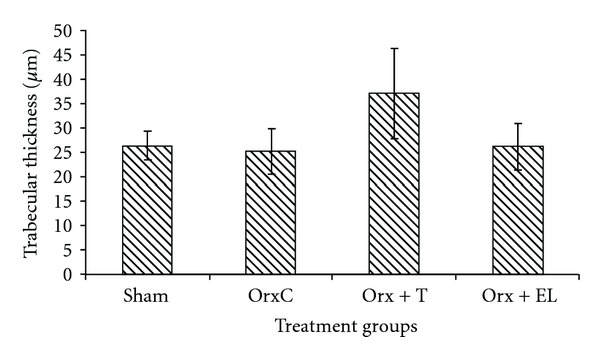
Mean trabecular thickness for all the groups. Data presented as mean ± SEM. There was no significant difference in trabecular thickness between the groups. Sham: sham-operated, OrxC: orchidectomised-control, Orx + T: orchidectomised and given testosterone replacement, Orx + EL: orchidectomised and supplemented with EL.

**Figure 11:**
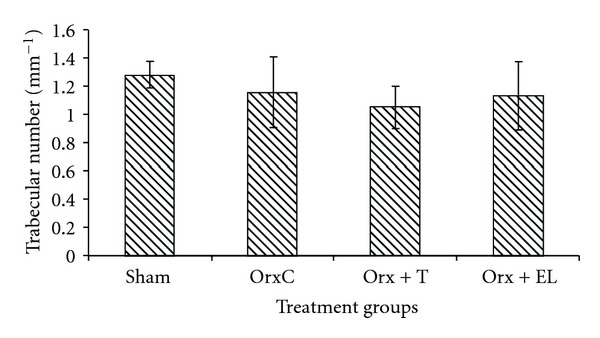
Mean trabecular number for all the groups. Data presented as mean ± SEM. There was no significant difference in trabecular number between the groups. Sham: sham-operated, OrxC: orchidectomised-control, Orx + T: orchidectomised and given testosterone replacement, Orx + EL: orchidectomised and supplemented with EL.

**Figure 12:**
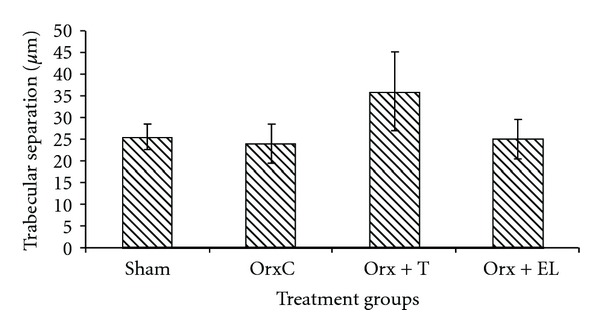
Mean trabecular separation. Data presented as mean ± SEM. There was no significant difference in trabecular separation between the groups. Sham: sham-operated, OrxC: orchidectomised-control, Orx + T: orchidectomised and given testosterone replacement, Orx + EL: orchidectomised and supplemented with EL.

There were no significant differences in the TbTh, TbN, and TbSp parameters between the different treatment groups (Figures 10, 11, and 12). 

## 4. Discussion 

In the past, neither alternative medicinal therapy received recognition in general nor herbal medicines accepted by pharmacological societies due to various reasons. Until recently, the effects of herbal products have not been standardised experimentally, and active compounds have not been isolated meticulously leading to gross dilution of the importance of alternative medicine. Several herbal-based products have shown therapeutic potentials in certain diseases such as malaria, cancer, and many others [17–19]. Recently, it has been reported that EL may prevent bone calcium loss in orchidectomised rats and has been shown as potential alternative or complimentary therapy for androgen-deficient osteoporosis [20]. EL has also shown the ability to activate 17 *α*-hydroxylase/17, 20 lyase (CYP17) enzyme that will increase testosterone level [28]. CYP17 is a P450 enzyme that catalyzes the last step of androgen biosynthesis in both the testis and the adrenals. 

In androgen-deficient patient (hypogonadism), there is a failure of the testis to produce physiological levels of androgen especially testosterone due to disruption of one or more levels of the hypothalamic-pituitary-testicular axis [1]. The choice of treatment for androgen deficiency syndrome is testosterone replacement therapy [1–4, 6]. Androgens play vital role in bone formation through androgen receptor (AR) which are present in most bone cells. Testosterone acts both direct and indirectly via the AR and following aromatization, via the estrogen receptor (ER), respectively [29–31]. Androgens may protect men against osteoporosis by maintaining the cancellous bone mass and expansion of the cortical bone [31].

However, the adverse effects of testosterone can be troublesome to the patients such as sleep apnea, polycythemia, liver toxicity, and more importantly, the risk of developing cancer [1, 5, 32, 33]. Hormone-dependent cancers such as metastatic prostate cancer and breast cancer may be stimulated during testosterone treatment. Testosterone is not recommended for those with underlying or high-risk factors for cancer [1, 5]. Therefore, an alternative to testosterone should be recommended to these patients. Based upon earlier studies, EL may increase the testosterone levels and preserve the bone calcium content [14, 20].

It was found that EL supplementation to normal male rats failed to elevate the testosterone levels compared to nontreatment rats. While for orchidectomised rat, the model for androgen-deficient osteoporosis [21], there was a significant reduction in the testosterone levels. Similar findings were found in several other studies which reported that the testosterone level falls immediately after orchidectomy [34, 35]. Testosterone replacement was shown to reverse the effect of orchidectomy. The testosterone levels of the orchidectomised rats remained low with EL supplementation (Figure 4). These findings showed that EL supplementation did not alter the testosterone levels of both normal and testosterone-depleted rats.

Based on the histomorphometric analysis of the first part of the study, there was no significant change in the BV/TV of normal rat with EL supplementation. In the second part of the study, the drop in the testosterone level in the OrxC group was associated with the reduction in BV/TV. When the testosterone level was raised with testosterone replacement in the Orx + T group, BV/TV was restored to that of the Sham group. Testosterone was able to improve the trabecular bone volume of orchidectomised rats; however, EL failed to emulate the testosterone action. This may be contributed by the failure of EL to raise the testosterone level of orchidectomised rats as that demonstrated by testosterone replacement. Only the BV/TV parameter produced significant findings. Although there were differences in the rest of the structural parameters, they failed to reach statistical significance among the groups. 

Clinical study in human has found that the dose up to 600 mg/kg did not cause any adverse effects [36]. EL is normally recommended to be administered by men at the dose of 200–400 mg daily. The dose of EL used in the present study is equivalent to the dose of about 100 mg/day in human, which is considered safe. Acute toxicity study revealed that the LD50 for EL extract was more than 5000 mg/kg [37]. It is unlikely to cause fatality in human as the equivalent dose of EL is 35 kg in a 70 kg man. In subacute toxicity study, EL extract at doses of more than 1200 mg/kg was shown to cause pathological changes in the rat liver. This dose was equivalent to approximately 8200 mg taken by a 70 kg man [37]. In the first part of the study, EL supplementation (EL group) did not seem to elevate the testosterone levels in rats with intact testes. This contradicted the finding that EL raised testosterone level in rats [8] and late onset hypogonadism patients which used different dosage [14]. However, there is a possibility that EL may only work when there is testosterone deficiency and that the testes are still intact. EL may only work to rectify the androgen deficiency state by stimulating the testes to produce testosterone. In androgen-deficient osteoporosis model, the rats were orchidectomised; therefore, we cannot determine if that was the case. However, it was proven that EL was unable to raise the testosterone level in rats with the absence of testes. 

## 5. Conclusion 

EL failed to emulate the testosterone replacement's ability to raise the testosterone level and restore the bone structure of orchidectomised rats. It also failed to produce any effect when supplemented to normal male rats. Further studies are required to determine if EL may be useful in androgen-deficient state if the testes are still intact.
